# Jumping up a level: Target distance and angle estimation facilitates successful landing in a jumping glass katydid

**DOI:** 10.1016/j.isci.2025.112738

**Published:** 2025-05-23

**Authors:** Shannon-Louise Harrison, Charlie Woodrow, Chloe K. Goode, Fernando Montealegre-Z, Denis Charles Deeming, Gregory P. Sutton

**Affiliations:** 1 University of Lincoln (UK) School of Life Sciences, LN6 7TS Lincoln, UK; 2 Department of Ecology and Genetics, Evolutionary Biology Centre, Uppsala University, Norbyvägen 18 D, 752 36 Uppsala, Sweden

**Keywords:** Zoology, Entomology, Biomechanics

## Abstract

Jumping is one of the most used forms of locomotion by insects, and a characteristic trait of the Orthoptera (locusts, crickets, and allies). Their specialized jumping behaviors have evolved for various functions, including travel, predator evasion, and flight initiation. While these jumping behaviors have been studied, targeted jumps required for navigating complex environments and hunting have received little attention. Here, we document a vertical jumping behavior in a species of neotropical predatory bush cricket (*Phlugis cf. celerinicta*. Tettigoniidae: Meconematinae), which uses visual cues to estimate target distance. Jumping kinematics were adjusted between jump heights (50mm, 75mm, and 100mm), with an increase in linear velocity and decrease in angular velocity at higher target heights. Body and leg postures also varied between jump heights. This study provides evidence that *P. celerinicta* can independently control both the speed at take-off and rotation rate based on target distance to achieve a precise and controlled landing.

## Highlights

• Jumping kinematics varied across different target heights in a predatory katydid
• Linear velocity increases and angular velocity decreases with target height
• Changes in body postures prior to take-off impacted the jump trajectories
• More energy was partitioned to translational movement as jump height increased

## Graphical abstract

**Figure:**
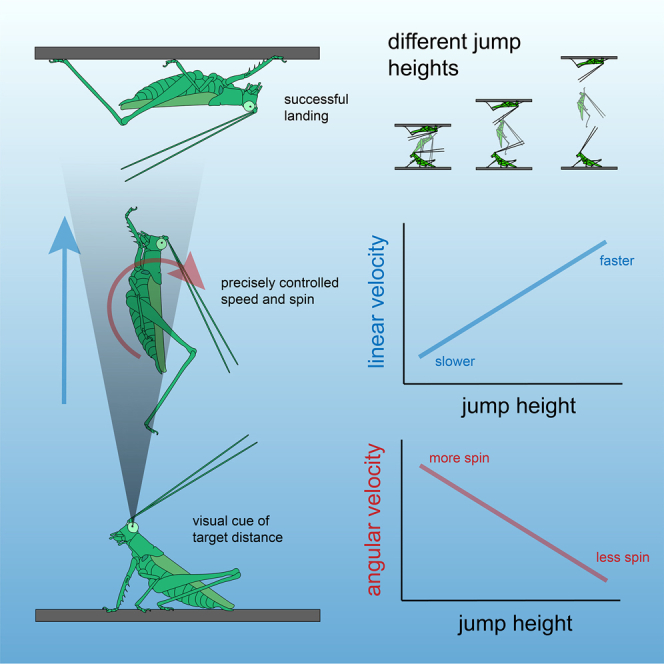

## Introduction

Orthopterans (grasshoppers, katydids, and crickets) are famously proficient jumpers, generating powerful leaps with precisely controlled speed and direction.^1^^,^^2^^,^^3^^,^^4^ Previous research into the jumping kinematics of this group has generally focused on jumps as an anti-predator response, using startling techniques to elicit escape behaviors.^2^^,^^4^^,^^5^^,^^6^^,^^7^ Jumping in other contexts, such as prey capture and habitat navigation, has received less attention. We demonstrate here that, during locomotory jumps, the predatory species, *Phlugis cf celerinicta* (Tettigoniidae: Meconematinae)^8^ precisely controls its speed and pitch rotation, allowing it to land on overhead targets of varied distance.

Jumping is a very effective means of locomotion, particularly useful when moving across long distances, evading predators, and initiating flight.^9^^,^^10^^,^^11^^,^^12^ A common consequence of jumping, particularly in orthopterans, is rotation during a jump due to asymmetry between forces acting on different parts of the body.^2^ Therefore, in orthopterans, which generate a lot of force from the hind limbs, the control (or lack) of spin when jumping can have a huge effect on their maneuverability.^2^^,^^13^ For escape jumps by juvenile locusts (*Schistocerca gregaria*), the percentage of energy devoted to spin is approximately constant^2^ but the jump kinematics are more variable,^5^ with little evidence for precise control.^2^^,^^11^ This is perhaps not surprising given that the animal is attempting to simply escape rather than to move to another chosen location. Despite the interest into the jumping mechanics of orthopterans, little research has investigated how spin may affect jumping in species that jump in the context of prey capture, where achieving a precise landing site is highly advantageous.

The Phlugidini (commonly known as glass katydids) are a widespread group of cryptic predatory katydids that use visual cues to aid in perception of target distance and prey capture.^8^^,^^14^ Unlike other neo-topical katydids, the Phlugidini are not fully nocturnal, but employ sit and wait tactics for prey capture during the day.^8^^,^^15^ This occurs by tracking the shadow of their prey beneath a semi opaque object such as a leaf. Prey acquisition occurs through a rapid jump to capture behavior whereby the glass katydid typically rotates its body about the leaf, capturing the prey object.^8^ Here, we present the first documentation of this behavior in a laboratory setting.

As with other katydids,^4^^,^^11^ *P. celerinicta* is a muscle-actuated jumper, which means that unlike spring driven jumpers^11^^,^^16^^,^^17^^,^^18^ the hind leg muscles are used to directly power the jumps demonstrated here. It has previously been suggested that the use of muscle actuation allows for finer control opposed to the ballistic propulsion generated by spring-actuated mechanisms.^4^^,^^11^^,^^13^ This level of control is not only reflected in the jumping kinematics but also the animal’s energy budget (how much energy an animal partitions between forward and rotational movement).^11^ Therefore, studying this specific jumping behavior will allow us to determine whether more precise control is achieved when using a muscle-actuated system in terms of linear and angular velocities across varying distances.

## Results

### Null hypothesis: Predicted relationship between linear and angular velocity

**Figure 1:**
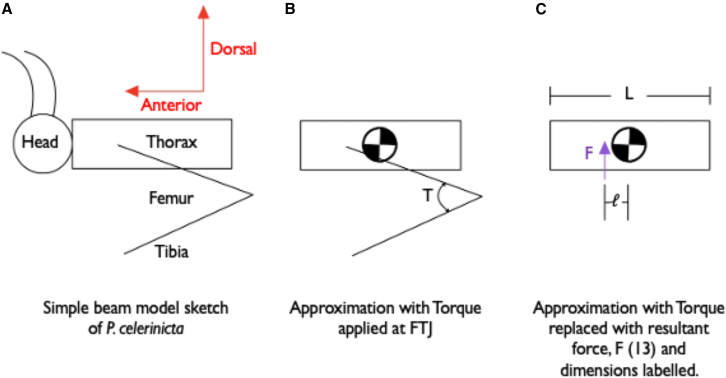
Simple illustration of a beam model depicting an insect prior to take-off This illustrates incrementally simpler models of an insect jumping to calculate the relationship between linear and angular velocity if each jump starts from an identical body position. (A) A mechanical sketch of the insect. (B) A simplified sketch modeling the head and thorax as a rectangular prism, with the muscle force in the legs acting as a torque, T, in the femorotibial joint (FTJ) (similar to the model in^13^). (C) A further simplified sketch modeling the force from the legs, F, applied at a distance, l, from the center of mass. The rectangular prism has a length L, and a mass m.

A mechanical analysis of the forces on the body during take-off provides a quantitative hypothesis for the relationship between take-off velocity and angular velocity during a jump. *P. celerinicta*, like all crickets, generates a jump by applying a torque about the FTJ (Figure 1B). This torque results in a vertical force, *F* (Figure 1), being applied at the body during the entire leg extension.^13^ This force is applied at a distance *l*, from the center of mass, resulting in a clockwise torque on the body via Newton’s second law $F=m\ddot{y}$ and via balance of moments $Fl=\frac{1}{12}mL^{2}\ddot{\theta }$. The angular acceleration of the body, $\ddot{\theta }\text{,}$ and linear acceleration of the body, $\ddot{y},\text{are}\;\text{thus}\;\text{tightly}\;\text{coupled}\;\text{with}\;\frac{\ddot{y}}{\ddot{\theta }}=\frac{L^{2}}{12\;l}$. Likewise, the angular and linear velocity are equally coupled by the same equation (this derivation is similar to that provided in^19^ for locomotion). Consequently, if a *P. celerinicta* increases jump height by increasing the force in the legs (and makes no other adjustments to its posture), angular velocity and linear velocity will increase by equal measure: linear velocity and angular velocity will be positively correlated.

### Kinematics

**Figure 2:**
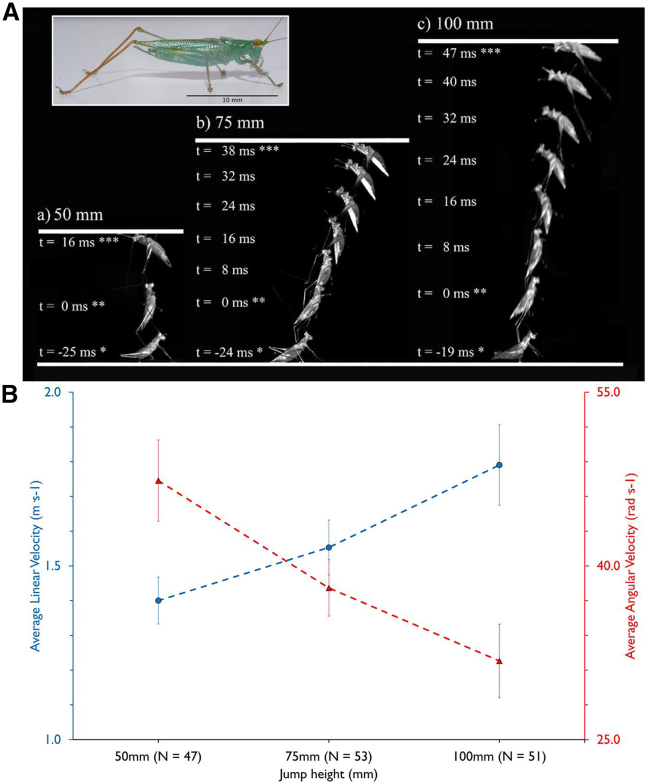
Jump progression stills and the resulting kinematics at take-off (A) Compilation of a series of still images of the jump progression of the *Phlugis celerinicta* at 50 mm, 75 mm and 100 mm (left to right) from at rest (indicated by ∗ in the photograph), during take-off (indicated by ∗∗) and at landing (indicated by ∗∗∗), filmed at 2,000 frames per second. (B) Mean (±SE) linear velocity (m·s^−1^; blue circles) and angular velocity (rad·s^−1^; red triangles) of the jumps at different jump heights (note the differences in the Y axes).

When presented with 3 overhead target heights (50 mm, 75 mm, and 100 mm above a base platform; Figure 2A) the glass katydids jumped faster as jump height increased (F_2,143.07_ = 49.412, *p* < 0.001) (Video S1). The average linear velocities (*lv*) were 1.40 (SE = 0.07), 1.55 (0.08) and 1.79 (0.12) m·s^−1^, for the progressively higher targets, respectively (Figure 2B, Table S1). Conversely, angular velocity (*av*) decreased (F_2,143.07_ = 27.681, *p* < 0.001) across the jump heights spinning at 47.36 (SE = 3.51), 38.10 (2.43) and 31.80 (3.18) rad·s^−1^, for the progressively higher targets, respectively (Figure 2B; Table S2). Therefore, as jump height increased, to accomplish the 180° (3.14 radians) rotation and orientate body position for a successful targeted landing there was an increase in linear velocity and a simultaneous decrease in angular velocity. If *P. celerinicta* were generating increased jump heights by only increasing muscle force in the leg, linear velocity and angular velocity would positively correlate (see Figure 1 and associated text). The negative correlation between angular velocity and linear velocity (Figure 2B) indicates that the *P. celerinicta* is both increasing the amount of force in the legs (to increase linear velocity) and changing posture (to decrease angular velocity).

Video S1. High speed jumps between target heights from take off to landing of *P. celerinicta*, related to STAR Methods

### Body positioning

In addition to being able to perceive the difference in distance between jump heights, the recorded jumps indicated that the animals were adjusting body position prior to take-off (Table S4). There were significant differences in body position, i.e., the distances between given points of the body when viewed laterally, between the experimental jump heights (Table S5). While stationary, there were no significant difference seen in the average FTJ (F_2,143.02_ = 0.753, df = 2, *p* = 0.473, Tables S4 and S5). However, at take-off, there was a significant increase in average FTJ angle between jump heights (F_2,143.07_ = 18.672, df = 2, *p* < 0.0001) ranging from 129 (±4.39) degrees at 50 mm to 144 (±2.65) degrees at 100 mm (Tables S4 and S5).

### Energetics

Of the 151 jumps measured here, there was a highly significant decrease in percentage of the energy budget allocated to rotation (F_2,143.07_ = 78.972, *p* < 0.001). At the jump height of 50 mm the insects allocated an average approximation of 2% of their energy to rotation, at 75 mm this decreased to 1% and 0.6% at 100 mm (Figure S2 and Table S3).

## Discussion

In this study, we demonstrated that a species of a predatory species of glass katydid could alter both its linear and angular velocities when presented with an increasingly higher target distance with a 100% success rate. The ability to control jump direction, speed, and body rotation has only previously been demonstrated in one other species of predatory invertebrate, the mantis *Stagomantis theophila*.^20^ *S. theophila* uses its forelimbs as counterweights at specific phases of the jump, which allows for some control of spin. However, when compared to a similarly sized mantis, in the glass katydid studied here, linear velocit*y (lv)* was approximately 1.5 times greater, even at the variably “slowest” jump height of 50 mm. Likewise, the glass katydid rotated 180° during a jump (compared to the mantis’s 45°) and did so with five times the angular velocity (*av*) of the mantis. Compared to an escape jump in another katydid (*Mecopoda elongata*), *lv* was similar but *av* was comparably faster in the glass katydid (5). Much like in the mantids, this is likely due to the difference in the motivation of the jumping behavior. *P. celerinicta* is an arboreal predatory species and so precise control of movement is not only vital for general movement about the treescape but also rapid prey capture within this complex environment.^11^ In order to perform a successful launch and attack, the glass katydid has to perform an entire inversion of its body during the jump, whereas during an escape jump (as demonstrated in *M. elongata*) precise landing may not be prioritized and, as such, spin is achieved passively opposed to actively. The success of these jumps is likely aided by the insects’ relatively large protruding eyes, with the praying mantis possessing a similar ocular morphology which provides them with acute depth perception.^21^

Generating an increased take-off velocity is relatively simple for this katydid, because increasing the muscle tension in the extensor tibiae muscle will translate into increased speed (Sutton and Burrows, 2008). By contrast, controlling angular velocity could be done in three, non-exclusive ways: (1) *P. celerinicta* could change the body position prior to the jump to give the animal less angular momentum at take-off (as mantises do (25)); (2) *P. celerinicta* could distribute angular momentum through limb movements in the air (as mantises also do); or (3) *P. celerinicta* could interact with wind resistance to alter spin in the air (as ants and springtails do^22^^,^^23^). Rotating limbs and engaging wind resistance both require the animal to be in the air long enough to engage these strategies; for example, the mantis limb rotation occurs after 50 ms in air, while the springtail wind resistance changes on rotation occur after 30–40 ms in air.^23^^,^^24^ Additionally, body mass and relative size may also have a large effect, with the relative effects of wind resistance increasing as organisms get smaller.^25^ The relatively reduced average time the *P. celerinicta* spends in the air during these jumps (from 20 ± 5.1 ms for the 50 mm jumps to 44 ± 7.7 ms for the 100 mm jumps) suggests that body position changes before the jump and as such is the primary way *P. celerinicta* is controlling its angular velocity. Moreover, for the 50-mm jumps, the rotational velocity at take-off was 47 ± 15.6 rad/s, while the average angular velocity across the entire jump was 47 ± 9.2 rad/s, suggesting that there were no changes to the *P. celerinicta*’s angular velocity while the animal was in the air. For the 100-mm jumps, the angular velocity at take-off was 41 ± 12.1 rad/s, while the average angular velocity across the whole jump was 31 ± 8.4 rad/s, suggesting that there may have been some changes in angular velocity while the animal was airborne. We suggest that changes in body position affecting the initial angular momentum at take-off is the primary way the insect is controlling angular velocity, although further study should be performed to determine if *P. celerinicta* is also controlling angular velocity through angular momentum manipulation of its limbs or aerodynamic interactions with the air.

Additionally, previous research into orthopterans’ jumping mechanics suggest that the disproportionately longer hind limbs allow for amplification of jump performance in both spring-actuated locusts^13^ and muscle-actuated bush crickets.^4^ *P. celerinicta* is no exception to this trend, with its hind legs being typically 1.37 times the length of its body. While stationary (prior to take-off), we found multiple differences in body position, however, there was no distinct parameter which helped to explain how the insects achieved such vast differences between linear and angular velocity at the varying target heights. However, as jump height increased, there was a significant increase in femorotibial joint angle (became more obtuse). In the escape jumps of adults of the dark bush cricket *Pholidoptera griseoaptera*, it was found that after the initial acceleration phase of the jump, once the legs left the ground there was negligible amount of angular rotation due to the upward projection of the legs.^4^ This allowed the insects to propel themselves forward, opposed to performing a backward spin as seen in similarly sized locusts (*S. gregaria)*.^2^ As to whether this was a controlled movement in the *P. griseoaptera* or simply an effect of ground reaction forces on the body post take-off is unknown. However, this observation does demonstrate how the placement of the legs at take-off, and consequently the rest of the jump, likely impacts jump trajectory.

In *P. celerinicta,* the reduced take-off leg angles at lower target heights are likely affecting the animals’ moment of inertia (a body’s ability to resist rotation due to the distribution of its mass^24^). When the hind legs are situated closer to the body at take-off (as is seen when the hind legs are at smaller angles), the animal’s moment of inertia is smaller, which in turn allows it to spin faster, as demonstrated in the targeted jumps performed at 50 mm compared with those seen at 100 mm. This is a common phenomenon seen in human figure skating in which the dancer will pull their arms inward in order to increase their rotation speed.^26^ The insects used in this study were all adults of similar size, and further investigation into how body proportions and mass distribution affect jumping ability at differing targeted heights would be valuable. This would be particularly beneficial in understanding how scaling differences across body size may impact an insect’s ability to control angular velocity, as seen in *S. gregaria* and *M. elongata*.^11^

Previous research suggests that there is a positive correlation between *lv* and *av* during escape jumps^2^^,^^11^ but the glass katydids studied here demonstrated that during targeted locomotory jumps, independent control of *lv* and *av* was possible. This was reflected in how the insects partitioned their energy when jumping. The decrease in energy partition allocated to rotating the body with an increasing target height is in direct contrast with to previous research wherein energy budgets between *S. gregaria* and *M. elongata*, were very similar despite these two orthopteran species using different jumping mechanisms.^5^^,^^11^ As previously discussed, this likely comes from the different jumping motivation between these studies. Within a muscle-actuated system, it has been suggested that while the system itself is subjected to greater size related constraints,^27^^,^^28^ the advantages of direction muscle actuation allows for more fine-tuned control, allowing the animal to make potential adjustments prior to or during movements.^4^^,^^11^^,^^17^ This plasticity in muscle control and coordination appears to be a proportionate response to the insects’ energy budget, highlighting the importance of jumping motivation when studying jumping kinematics. In the future, it would be very interesting to explore whether this targeted jumping behavior could be replicated in a spring-actuated system and whether the kinematics would align with the more finely tuned motor control or that of the relatively faster, less controlled jumps previously demonstrated in other spring-actuated systems.

This study presents the first account of independent control of linear and angular velocity during an orthopteran jump. Further work should aim to identify whether this behavior is unique to the Phlugidini or more widespread across Orthoptera and insects more generally.

### Limitations of the study

As with many experimental conditions, the jumps recorded here were in a controlled laboratory setting and, as such, may not entirely reflect the insects’ true capability in a potential more complex environment. For example, the jumps recorded had a 100% success rate upon landing, which may not be completely representative of jumps seen by this species in the wild. Additionally, the controlled conditions mean that factors which may have impacted the jump trajectories, such as variability int substrate compliance, atmospheric differences, and visual cues were not considered here. Furthermore, the individuals studied here were approximately the same body mass and size and as such these jumps may only be reflective of individuals at this specie instar—we do not know if these accurate targeted jump trajectories are reflective of physiology, age, and experience.

## Resource availability

### Lead contact

If there are any further questions or request for resources, please direct them to the lead contact, Shannon Harrison (ShHarrison@lincoln.ac.uk).

### Materials availability

No new materials were generated in this study.

### Data and code availability

• The data generated from this study is available in a Github repository (see key resources table).
• The code used for the analyses can also be found in the Github repository.
• Any additional information required to reanalyze the data reported in this paper is available from the lead contact upon request.

## Acknowledgments

We would like to thank the Royal society for funding this project (UF120507). CK Goode and GP Sutton were supported by the UKRI Grant Number (MR/T046619/1) awarded to GPS as part of the NSF/CIHR/DFG/FRQ/UKRI-MRC Next Generation Networks for Neuroscience programme. Additionally, we would like to thank Dr Fabio Sarria and Mr Lewis Holmes for aiding in collection of the animals and maintenance of them in the lab while experiments were being conducted. We also appreciated the kind feedback received from Dr Delyle Polet whose discussion and ideas helped us to refine this study.

## Author contributions

S.L.H., C.W., and G.P.S., conceptualization of the study. S.L.H. and C.W., experimental data collection. S.L.H., data analyses and kinematic calculations. S.L.H., C.W., and C.K.D., statistical analysis and R markdown document curation. C.K.G., figure design and creation. C.W., design and creation of the graphical abstract cover photo submissions. S.L.H. creation of supplemental video. F.M.-Z. provided the insects. S.L.H. drafted initial manuscript and then all authors implemented revisions.

## Declaration of interests

All authors declare no conflicts of interest.

## STAR★Methods

### Key resources table

**Table:** 

REAGENT or RESOURCE	SOURCE	IDENTIFIER
**Deposited data**		

Repository data and R code	This study	https://github.com/shannonharrison98/Jumping-up-a-level-Target-distance-

**Experimental models: Organisms/strains**		

Glass katydid (*Phlugis cf. celerinicta)*	Minster house insectary (Poplar Ave, Lincoln LN6 7DL). 7 individuals (2 female and 5 males).	N/A

**Software and algorithms**		

R studio (Version 2024.12.0+467)	R Development Core Team^29^	https://posit.co/downloads/

### Experimental model and study participant details

Seven individuals were studied (5 males, 2 females), with a body mass range of 0.0678 – 0.0822 g. Individuals were housed in Minster house insectary (Lincoln, LN6 7DL). Individuals were housed in different sexed pairs (where possible) and were kept in incubators which were set to 25 degrees Celsius which ran a 12/12 light/dark cycle. They were fed every 2 days on a diet of apples, dry dog food and pollen.

### Method details

#### Jumping methodology and camera set-up

Jumps were recorded using the Photron FastCam Mini at 2000 frames per second with a shutter speed of 1/6000. Within the filming area (3000 mm^3^ glass tank), the animals were placed on a wooden block which was situated orthogonally to the camera. Laterally attached to the block was a ruler which acted as a scale bar during video analysis. Additionally, the varying jump heights were attached to the rightmost edge of the block, each of which had a piece of balsa wood attached to the top of the jump height so that the balsa wood ran parallel to the wooden block. On the underside of these balsa wood targets was a 15 mm black dot which represented a pseudo prey item ^.^ The specimens were encouraged to jump by placing a paintbrush end in front of its face before slowly lifting this paintbrush upwards, so that the animal’s tracked its movement until their attention was drawn to the target dot above. Prior to jumping, *P. celerinicta* exhibits a motion parallax behaviour in which the fore and mid legs are used to sway the body side to side.^30^^,^^31^ Jumps were only classed as a targeted jump if this behaviour was seen. The final dataset consisted of 151 recorded jumps from seven animals: 47 jumps to the 50 mm target, 53 jumps to the 75 mm target, and 51 jumps to the 100 mm target. Of these 151 jumps, 100% of them landed successfully overhead, i.e., landed on the ceiling after rotating through 180 degrees and not falling off (see Video S1).

#### Jump analysis

The recorded jumps were digitised using Tracker.^32^ Length measurements (Body, femur and tibia) lengths were taken using Tracker. Body length was defined from the base of the antenna to the most distal point of the abdomen. Hind leg length was split between two measurements; femur length (mm) and tibia length (mm) (Figure S1) and then summed for total leg length. To map body position throughout the jump, four points of the animal were tracked (Figure S1), with each frame of the jump tracked from the stationary position to the inverted landing. From this dataset, the x, y coordinates of the stationary, take-off (defined as when both feet leave the ground) and contact to target were extracted.

#### Kinematic and energetics calculations

From the coordinate data generated above, linear, and angular velocity were calculated from take-off to contact as follows:

$$Linear\;velocity\;\left(m\cdot s^{-1}\right)=\sqrt{\left(\frac{{dx}^{2}}{dt}\right)+\left(\frac{{dy}^{2}}{dt}\right)}$$

$$Angular\;velocity\;\left(rad\cdot s^{-1}\right)=\left(\frac{d\theta }{dt}\right)$$

$$Inertia\;\left(kg\;\cdot \;\right)m^{2}=\frac{1}{12}\left(m\;\cdot \;{body\;length}^{2}\right)$$

$$Translational\;kinetic\;energy=\frac{1}{2}m\;\cdot \;v^{2}$$

$$Rotational\;kinetic\;energy=\frac{1}{2}I\;\cdot \;{\dot{\theta }\;}^{2}$$

where *d* = displacement (m), *m* = body mass (kg), *t* = times (s), θ = body angle (radians), *I* = Inertia (kg·m^2^).^2^^,^^11^ For each jump the difference in x and y position of the frames of interest (stationary, take-off and landing) were calculated and then divided by the number of frames between each interest point. Thus representing and average velocity.

### Quantification and statistical analysis

Statistical analyses were performed in RStudio.^29^ For each parameter of interest linear mixed-effects models (LMMs) were ran using the lmer function^33^ whereby the response variable (s) was the parameter of interest, i.e., linear velocity and the explanatory (fixed) variable was jump height. Results of LMMs were assessed through type III ANOVAs using the Satterthwaite method. There were multiple data points for each animal, so to control for repeated sampling we treated the individual animal as a random factor in each model. In order to further test which groups were significantly different from one another, post hoc tests were performed using ‘emmeans’^34^ for each given parameter.

## Supplemental information

Document S1. Figures S1 and S2 and Tables S1S5
