# Supplementary material for: Jumping up a level: Target distance and angle estimation facilitates successful landing in a jumping glass katydid
Source: iScience. 2025 May 23;28(6):112738. doi: 10.1016/j.isci.2025.112738 (PMC12177184; doi:10.1016/j.isci.2025.112738)
Supplement: Document S1. Figures S1 and S2 and Tables S1S5 [file mmc1.pdf]

iScience, Volume *28*

Supplemental information

Jumping up a level: Target distance and angle estimation facilitates successful landing in a jumping glass katydid

Shannon-Louise Harrison, Charlie Woodrow, Chloe K. Goode, Fernando Montealegre-Z, Denis Charles Deeming, and Gregory P. Sutton

Supplemental items:

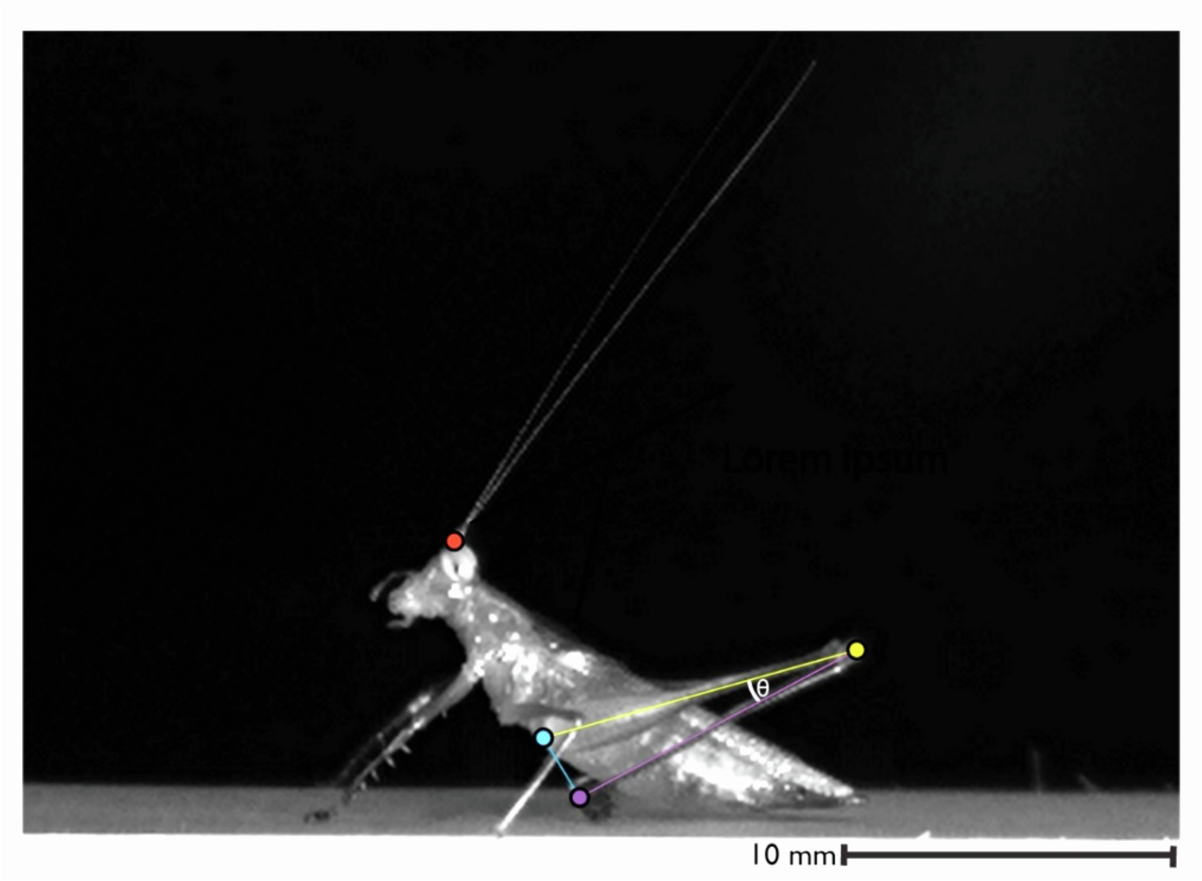

Figure S1. Illustrative diagram outlining the points tracked on the insects (Related to STAR methods).

Points tracked as follows; (circles; red = base of the antennae, blue = proximal point of the midline of the femur (PF), yellow = femoro-tibial joint , purple = proximal part of the foot (F). The lines represent the distance between the points, calculated using the x,y coordinates of each point. θ = the angle between the femur and tibia.

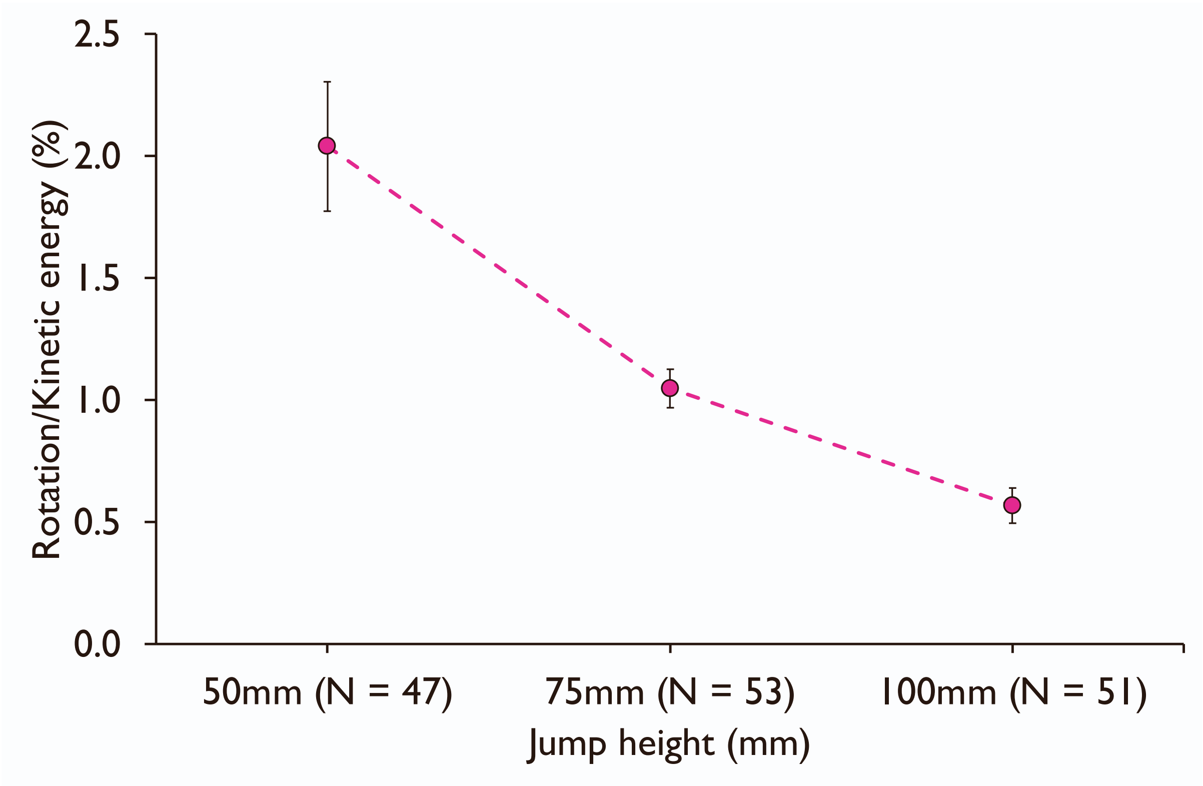

Figure S2. Energy budget plot across jump heights (Related to STAR methods).

Mean (± SE) percentage of energy attributed to rotation between jump heights.

Table S1. Pairwise t test for linear velocity across jump heights.

Output from a t test for pairwise comparisons (test statistic and p-value) comparing the average linear velocity between jump heights.

Jump height (mm)	100	50
50	-10.22, $p < 0.0001$	-
75	-6.71, $p < 0.0001$	-3.85, $p = 0.0002$

Table S2. Pairwise t test for angular velocity across jump heights

Output from a t test for pairwise comparisons (test statistic and p-value) comparing the average angular velocity between jump heights.

Jump height (mm)	100	50
50	7.34, $p < 0.0001$	-
75	2.63, $p = 0.0096$	4.94, $p < 0.0001$

Table S3. Summary of average hind leg dimensions from tracked data

Calculated Mean (± SE) of the hind leg dimensions from the tracked co-ordinate data. H = base of the antenna, PF = proximal femur, FTJ = femoro-tibial joint and F = Foot. The arrow denotes the calculated distance between these two points. Angles are reported degrees and the distances in mm.

Hind leg dimensions	50 mm	75 mm	100 mm
Stationary FTJ angle	116 (± 4.39)	120 (± 4.39)	120 (± 4.43)
Take-off	129 (± 2.62)	136 (± 2.61)	144 (± 2.65)
H → PF	21.4 (± 0.58)	22.4 (± 0.58)	23.9 (± 0.58)
H → FTJ	24.0 (± 0.59)	25.3 (± 0.59)	25.9 (± 0.60)
H → F	24.1 (± 0.69)	25.1 (± 0.69)	26.8 (± 0.70)
PF → F	2.91 (± 0.16)	2.91 (± 0.16)	3.01(± 0.16)
PF → FTJ	7.86 (± 0.21)	8.33 (± 0.21)	8.12 (± 0.22)
FTJ → F	4.34 (± 0.35)	7.24 (± 0.35)	6.95 (± 0.36)

Table S4. Anova output from tracked hind leg dimensions across different jump heights. Output from an ANOVA test with jump height as a factor. Dimensions were measured using tracked co-ordinate data between 4 points; H = base of the antenna, PF = proximal femur, FTJ = femoro-tibial joint and F = Foot. The arrow denotes the distance between these two points. Please see Figure S1 for a schematic representation of these points. Following post hoc tests at 0.05 significance level : [a] = significant difference in dimensions between 50-75 mm, [b] = significant difference between 50-100 mm and [c] = significant difference between 75-100 mm.

Hind leg dimensions	F value	Degrees of freedom	Residual Degrees of Freedom	P value
Stationary FTJ angle	0.7526	2	143.02	0.473
Take-off	18.672	2	143.07	<0.0001 [a,b,c]
H → PF	10.202	2	144.17	<0.0001 [b,c]
H → FTJ	11.102	2	142.65	<0.0001 [a,c]
H → F	10.928	2	143.82	<0.0001 [b,c]
PF → F	0.4480	2	143.66	0.6398
PF → FTJ	2.2477	2	143.47	0.1094
FTJ → F	58.676	2	143.71	<0.0001 [a,b]

Table S5. Pairwise t test for energy budget across jump heights

Output from a t test for pairwise comparisons (test statistic and p-value comparing the average energy attributed to rotation between jump heights.

Jump height (mm)	100	50
50	12.07, $p < 0.0001$	-
75	3.11, $p = 0.0023$	9.28, $p < 0.0001$
